# Predictors of outcomes following double-row rotator cuff repair: an assessment of all-suture or solid medial row anchor utilization at a single high-volume institution

**DOI:** 10.1016/j.xrrt.2025.100639

**Published:** 2025-12-11

**Authors:** Anna E. Crawford, Eric A. Mussell, Matthew P. Ithurburn, Brook Ostrander, David Brockington, Cristian Arceo, Glenn S. Fleisig, Marcus A. Rothermich, Michael K. Ryan, Benton A. Emblom, Jeffrey R. Dugas, E. Lyle Cain

**Affiliations:** a Andrews Sports Medicine and Orthopaedic Center, Birmingham, AL, USA; b The American Sports Medicine Institute, Birmingham, AL, USA; c School of Health Professions, University of Alabama at Birmingham, Birmingham, AL, USA; d Prevea Health Orthopaedics and Sports Medicine, Green Bay, WI, USA

**Keywords:** Rotator cuff repair, Double-row suture bridge, Solid anchor, Suture anchor, Arthroscopy, Patient-reported outcomes

## Abstract

**Background:**

Use of all-suture soft anchors in arthroscopic rotator cuff repair (RCR) has been shown to provide both biomechanical and functional advantages. However, predictors of clinical outcomes following RCR using all-suture anchors have not been well established. This study aimed to examine predictors of clinical outcomes following double-row suture bridge RCR using either all-suture or solid medial row anchors.

**Methods:**

We retrospectively identified patients at our institution who underwent arthroscopic RCR. Patients were eligible for inclusion if they underwent primary arthroscopic RCR using a double-row suture-bridge technique with either all-suture or solid medial row anchors, were between the ages of 18 and 85, and were at least 2 years postoperative. We collected demographic, clinical, and intraoperative data via electronic health record review. Patient-reported outcomes were evaluated at follow-up using the American Shoulder and Elbow Surgeons (ASES) Standardized Shoulder Assessment and visual analog scale (VAS). Proportions meeting Patient Acceptable Symptomatic State (PASS) thresholds for each were calculated. Within either anchor group, we used univariable linear and logistic regression to examine predictors of scores and meeting PASS thresholds at follow-up, respectively.

**Results:**

In total, 352 patients completed follow-up (mean age = 60.3 ± 10.0 years; 61% male; mean follow-up time = 3.0 ± 0.8 years). Within the all-suture anchor group (n = 280), male sex (*P* = .04) and longer follow-up time (*P* < .01) were associated with improved ASES scores, higher odds of meeting the PASS cutoff for the ASES (*P* < .01), improved VAS scores (*P* = .01), and higher odds of meeting the PASS cutoff for the VAS (*P* = .02). Within the solid anchor group (n = 72), large tears were associated with worse ASES scores (*P* < .01), lower odds of meeting the PASS cutoff for the ASES (*P* = .02), and worse VAS scores (*P* < .01. Longer follow-up time was associated with higher odds of meeting the PASS cutoff for the VAS (*P* = .04).

**Conclusion:**

Following arthroscopic double-row suture-bridge RCR, longer follow-up time was associated with better patient-reported outcomes (PROs) in both anchor type groups. However, smaller tear size was associated with better PROs only within the solid anchor group, whereas male sex was associated with better PROs only within the all-suture anchor group.

In the past two null decades, rotator cuff repair (RCR) surgical techniques have undergone significant advancements, particularly in arthroscopic instrumentation and suture anchor technology. Arthroscopic RCR has become standard practice, offering benefits such as reduced pain, decreased deltoid dysfunction, and improved early recovery of range of motion compared to mini-open RCR.^38^ Among recent advancements in arthroscopic RCR is the introduction of all-suture anchors, which have garnered attention for their potential advantages over traditional solid anchors. In general, all-suture anchors are manufactured with smaller diameters compared to solid anchors, allowing for a greater number of anchors to be placed and mechanical load to be more evenly distributed, with some studies demonstrating diminished bone displacement during both drilling and setting of the anchors, offering the potential to conserve bone stock.^5^^,^^22^^,^^41^ In particular, this may be most advantageous in the setting of revision RCR, when preserving bone stock is most critical. In addition, all-suture anchors may mitigate certain complications reported to occur following RCR with conventional solid anchors, such as migration, synovitis, chondrolysis, reactive osteolysis, and chondral damage from third-body wear.^5^^,^^8^^,^^10^^,^^14^ The use of all-suture anchors has been associated with reduced bone reaction and perianchor cyst formation compared to polyetheretherketone anchors and bioabsorbable anchors following arthroscopic RCR; potentially implying a more dependable postoperative biologic fixation.^17^^,^^35^ This holds notable clinical significance, given that there is evidence establishing a correlation between the severity of bone reaction, the development of perianchor cysts, and rotator cuff retear.^35^ Lastly, use of all-suture anchors is less technically demanding than more traditional anchors, as use of a cannulated drill guide replaces the need of recreating the trajectory of the preparation angle when inserted the anchor.^41^

Advances in suture anchor technology have also improved RCR by introducing anchors with the ability for each individual suture anchor to integrate independently from other anchors. Capitalizing on this new capability, the transosseous equivalent or suture-bridge repair technique incorporates suture limbs from a medial row anchor into a lateral row, theoretically aiding healing by compressing the tendon to the rotator cuff footprint.^9^ Recent studies show double-row suture-bridge RCR technique demonstrates improved healing when compared to a single-row approach.^28^^,^^39^ In addition, biomechanical comparisons between all-suture and solid anchors demonstrate similar pullout strength, but with inconsistent consensus regarding variability among different all-suture anchors available on the market.^2^^,^^3^^,^^11^^,^^22^^,^^25^^,^^36^^,^^42^ Application of an all-suture medial row in RCR shows similar mechanical performance and clinical efficacy, especially in large or massive tears, favoring the double-row suture-bridge technique for optimal structural healing.^3^^,^^6^^,^^23^^,^^28^^,^^39^^,^^43^

Numerous factors are important in determining outcomes following RCR, some of which can be controlled by the surgeon and clinical team, and others that cannot. Among these, key factors under control of the clinical team include operative technique, and more specifically, the type and number of implant used. However, factors such as injury and demographic characteristics, including tear size, patient age, and patient sex, are outside the control of the clinician. The purpose of our study was to examine predictors of outcomes following double-row suture-bridge RCR using either all-suture anchors or solid anchors for the medial row.

## Methods

**Figure 1:**
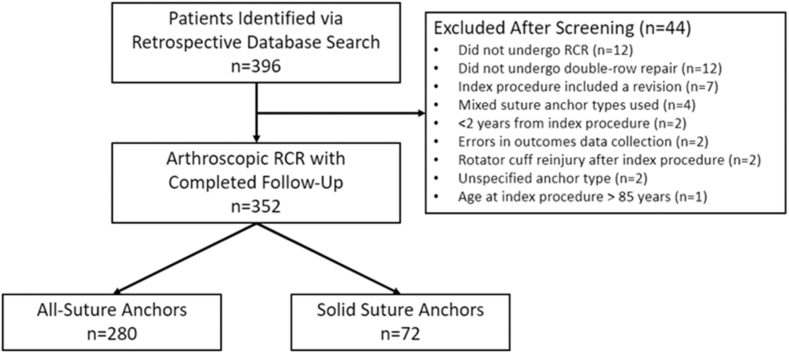
CONSORT diagram. *RCR*, rotator cuff repair; *CONSORT*, Consolidated Standards of Reporting Trials.

Prior to beginning this study, we obtained approval from the institutional review board at our institution (IRB protocol #6280-001). Following this, we performed a retrospective search of RCRs through the use of a billing-code database, featuring those procedures performed by one of 4 fellowship-trained orthopedic surgeons at Andrews Sports Medicine and Orthopaedic Center between 2016 and 2018. Potential patients were included according to the following criteria: (1) if they underwent primary, double-row, suture-bridge RCR using either solid or all-suture medial row anchors; (2) were between the ages of 18 and 85 years at the time of surgery; and (3) were at least 2 years postoperative at the time of outcomes data collection. Conversely, patients were excluded if they: (1) did not undergo double-row RCR; (2) required a subsequent revision RCR; (3) underwent an RCR procedure featuring a combination of solid and all-suture medial row anchors; or (4) suffered an isolated subscapularis tear or repair (Fig. 1).

### Patient selection, surgical technique, and postoperative rehabilitation

Patients were identified and selected for surgery if they failed conservative treatment (in the case of tears with an atraumatic etiology), or if they suffered a traumatic tear meeting operative criteria as determined by surgeon's discretion and clinical experience. All patients selected for RCR surgery had either a high-grade partial-thickness tear (greater than 50% thickness; determined intraoperatively) or a complete, full-thickness tear. Activity level, the presence or absence of degenerative glenohumeral changes, and Goutallier grade (indicative of the degree of fatty muscle degeneration) were also considered by the surgeon prior to selecting patients for RCR.^12^ In terms of the latter factors, surgical eligibility was contingent upon absent degenerative glenohumeral changes on radiographs and Goutallier grade less than 3.^12^

All RCR surgeries were performed arthroscopically. In addition to RCR, acromioclavicular arthritis was treated with distal clavicle excision, and pathologies involving the long head of the biceps tendon were treated with mini-open subpectoral biceps tenodesis when applicable. For each repair, the tear site was prepared for repair prior to use of a medial row transosseous equivalent (suture bridge) technique to reapproximate the rotator cuff to its original anatomic footprint, as described previously.^20^ The choice of solid or all-suture anchor use for the medial row was based on individual surgeon preference. Solid anchors were used for the lateral row (one or two 5.5-mm SwiveLock anchors; Arthrex, Naples, FL, USA). In the case of RCRs featuring use of all-suture medial row anchors, tears found to be large or massive were medialized slightly to within the margins of the articular cartilage. In the case of “L”-shaped or “U”-shaped tears, additional surgical techniques were utilized to provide optimal reduction, as previously described, including margin convergence, shoe lace repair, or other tendon-to-tendon repair and closure techniques.^20^

Postoperatively, all patients underwent an institutionally developed rehabilitation protocol, with specific physical therapy protocols for each tear size (small, medium, large, or massive). This process of rehabilitation began with early progressive passive range of motion exercises, focusing on regaining normal shoulder range of motion began on postoperative day 1. In the early phase of rehabilitation, an abduction sling was used for shoulder immobilization when not performing physical therapy exercises for 6 weeks, followed by the initiation of isometric strengthening exercises and continued focus on passive range of motion. Beginning at postoperative week 6, the second phase of rehabilitation focused on a progression of strengthening, active range of motion exercises, and functional activities of the shoulder and upper extremity. Monitored physical therapy with an additional home exercise program was continued for 12 weeks postoperatively. Return to limited activity was allowed after 12 weeks, with return to full, unrestricted activity at 24 weeks.

### Clinical and outcomes data collection

For patients determined to meet eligibility criteria for inclusion in the study, demographic data, clinical chart data, and operative note data were obtained from within our electronic health record. Data reviewed and collected included: age, sex, rotator cuff tear size, type of medial row anchors utilized, number of medial row anchors placed, and whether the patient underwent subsequent revision surgery for retear at our institution. Postoperative imaging was not performed on a routine basis, neither as part of clinical follow-up nor for the purposes of this study. In terms of tear size, rotator cuff tears were categorized as small (<1 cm), medium (1-3 cm), large (3-5 cm), or massive (>5 cm).^4^ Patient-reported outcomes data were collected following enrollment of RCR patients into a continually updated data repository (OBERD; Universal Research Solutions, Columbia, MO, USA), which distributed outcomes surveys electronically to those patients via automated email and/or SMS message at 2 years post-RCR. Patients who did not respond to these electronically distributed surveys were contacted via telephone and asked to complete surveys verbally. All patients provided either written or verbal informed consent prior to enrollment in our data repository or completion of any verbal surveys.

The questionnaire used to evaluate shoulder function was the American Shoulder and Elbow Surgeons (ASES) Standardized Shoulder Assessment Form. The ASES is a 100-point scale which aims to measure limitations or lack thereof in shoulder-related function and includes questions related to pain, instability, and ability to perform activities of daily living.^34^ The ASES has been shown to be a valid, reliable, and responsive method of assessing shoulder function in patients with rotator cuff pathology and following RCR and has a minimal clinically important difference value of 6.4 points.^24^ The ASES also includes a visual analog scale (VAS) for shoulder-related pain, which was used in the present study as an additional means of quantifying shoulder pain in our patients. VAS scores were scaled from 0 (no pain) to 10 (extreme pain).

### Statistical analyses

Summary statistics were calculated for baseline and follow-up demographic, clinical, surgical, and outcomes data within the entire cohort as well as within each medial row anchor group (all-suture; solid). For ASES and VAS scores at follow-up, we calculated the proportions of patients meeting the Patient Acceptable Symptomatic State (PASS) cutoffs previously established for RCR (ASES ≥ 78.0; VAS ≤ 1.7).^15^ Within the all-suture anchor group and the solid group separately, we examined predictors of follow-up questionnaire scores (ASES and VAS) and meeting PASS thresholds for the ASES and VAS at follow-up using univariable linear regression and univariable logistic regression, respectively. All assumptions for parametric statistical testing were evaluated and were met. For all analyses, the threshold of statistical significance was a *P* value <.05. All analyses were performed using IBM SPSS version 28.0 (IBM Corp., Armonk, NY, USA).

## Results

### Cohort demographics

A total of 396 patients were identified as potentially eligible through the retrospective database search, and outcomes data were successfully collected in 352 patients (89%) with a minimum of 2 years of follow-up (mean follow-up time = 3.0 ± 0.8 years) (Fig. 1). Full demographic, clinical, and surgical data for the entire cohort are presented in Table I. Of these patients, 280 (80%) patients had RCR performed using all-suture anchors for the medial row, and 72 (20%) had RCR performed using solid anchors for the medial row. Comparing the all-suture and solid anchor groups, there were no significant differences in age at surgery, age at the time of follow-up, average follow-up time, sex distribution, rotator cuff tear size, nor the number of medial row anchors used in each repair (all *P* > .05; Table II). In addition, there were no significant differences in follow-up ASES scores, VAS scores, nor the proportions meeting PASS cutoffs for the ASES and VAS (all *P* > .05; Table II).

**Table I:** Demographic, clinical, and outcomes data for the entire cohort (n = 352).

Variable	Entire included cohort (n = 352)
Age at surgery∗, yr	60.3 ± 10.0 Range (21.6, 80.9)
Age at follow-up∗, yr	63.1 ± 10.3 Range (25.1, 85.2)
Follow-up time∗, yr	3.0 ± 0.8 Range (1.9, 4.7)
Sex/sex, n (%)	138 F (39.2%) 214 M (60.8%)
RTC tear size†, n (%)	180 small (51.1%) 75 medium (21.3%) 49 large (13.9%) 48 massive (13.6%)
Number of medial row anchors, n (%)	1 anchor: n = 67 (19.0%) 2 anchors: n = 256 (72.7%) 3 anchors: n = 29 (8.2%)
ASES score at follow-up∗	89.0 ± 16.9 Range (10.0, 100.0)
VAS score at follow-up∗	1.2 ± 2.1 Range (0, 10)
Proportion of cohort meeting PASS cutoff for ASES‡	n = 287 (81.5%)
Proportion of cohort meeting PASS cutoff for VAS§	n = 268 (76.1%)

*F*, female; *M*, male; *RTC*, rotator cuff; *ASES*, American Shoulder and Elbow Surgeons; *VAS*, visual analog scale for pain; *PASS*, Patient Acceptable Symptomatic State.

∗ Data are presented as mean ± standard deviation.

† RTC tear size (small: <1 cm; medium: 1-3 cm; large: 3-5 cm; massive: >5 cm).

‡ PASS value for ASES (^3^ 78.0^15^).

§ PASS value for VAS (^3^ 1.7^15^).

**Table II:** Demographic, clinical, and outcomes data by solid or soft anchor groups.

Variable	Solid anchor group (n = 72)	All-suture anchor group (n = 280)
Age at surgery∗, yr	59.8 ± 9.3 Range (41.1, 74.8)	60.4 ± 10.2 Range (21.6, 80.9)
Age at follow-up∗, yr	63.4 ± 9.4 Range (45.5, 78.9)	63.2 ± 10.3 Range (25.1, 85.2)
Follow-up time∗, yr	3.58 ± 0.67 Range (1.98, 4.34)	2.84 ± 0.77 Range (2.00, 4.65)
Sex/sex distribution, n (%)		
Female	30 (41.7%)	108 (38.6%)
Male	42 (58.3%)	172 (61.4%)
RTC tear size†, n (%)	35 small (48.6%) 18 medium (25.0%) 11 large (15.3%) 8 massive (11.1%)	145 small (51.8%) 57 medium (20.4%) 38 large (13.6%) 40 massive (14.3%)
Number of medial row anchors, n (%)		
1 anchor	n = 17 (23.6%)	n = 50 (17.9%)
2 anchors	n = 48 (66.7%)	n = 208 (74.3%)
3 anchors	n = 7 (9.7%)	n = 22 (7.8%)
ASES score at follow-up∗	89.6 ± 17.8 Range (21.6, 100.0)	88.8 ± 16.7 Range (10.0, 100.0)
VAS score at follow-up∗	1.1 ± 2.1 Range (0, 10)	1.2 ± 2.1 Range (0, 10)
Proportion of cohort meeting PASS cutoff for ASES‡	n = 61 (84.7%)	n = 226 (80.7%)
Proportion of cohort meeting PASS cutoff for VAS§	n = 58 (80.6%)	n = 210 (75.0%)

*RTC*, rotator cuff; *ASES*, American Shoulder and Elbow Surgeons; *VAS*, visual analog scale for pain; *PASS*, Patient Acceptable Symptomatic State.

∗ Data are presented as mean ± standard deviation.

† RTC tear size (small: <1 cm; medium: 1-3 cm; large: 3-5 cm; massive: >5 cm.

‡ PASS value for ASES (^3^ 78.0^15^).

§ PASS value for VAS (^3^ 1.7^15^).

### Patient-reported outcomes: entire cohort and within anchor groups

For the entire cohort of 352 patients who completed follow-up, the average ASES score at follow-up was 89.0 ± 16.9, with 287 (82%) meeting the ASES PASS cutoff. The average VAS score for the entire cohort was 1.2 ± 2.1, with 268 (76%) patients meeting the VAS PASS cutoff (Table I). Within the all-suture anchor group (n = 280), the average ASES score at follow-up was 88.8 ± 16.7, with 226 (81%) meeting the ASES PASS cutoff. For the all-suture anchor group, the average VAS score was 1.2 ± 2.1, with 210 (75%) patients meeting the VAS PASS cutoff (Table II). Within the solid anchor group (n = 72), the average ASES score at follow-up was 89.6 ± 17.8, with 61 (85%) meeting the ASES PASS cutoff, and the average VAS score for the entire cohort was 1.1 ± 2.1, with 58 (81%) patients meeting the VAS PASS cutoff (Table II). There were no group differences in average ASES score, average VAS score, nor the proportions of patients meeting PASS criteria for either the ASES or VAS (all *P* > .05; Table II).

### Predictors of outcomes: all-suture anchor group (n = 280)

Examining predictors of ASES outcomes at follow-up within the all-suture anchor group only, we found that male sex and longer follow-up time were associated with higher (better) ASES scores (R^2^ = 1.6%, *P* = .04; R^2^ = 2.6%, *P* < .01; respectively). In addition, we found that longer follow-up time was also associated with higher odds of meeting the ASES PASS cutoff (odds ratio [OR], 2.3, *P* < .01). Specifically, for every 1-year increase in follow-up time, there was approximately 2.3 times the odds of meeting the ASES PASS cutoff. Whereas we found that male sex demonstrated a statistical trend towards association with increased odds of meeting the ASES PASS cutoff (OR, 1.79, *P* = .06), this did not reach statistical significance. Mean ASES scores and the proportions meeting the ASES PASS cutoff by sex are shown in Supplementary Appendix S1 (Supplemental Data). Tear size, age at surgery, age at follow-up, and the number of medial row anchors were not significantly associated with either ASES score nor the odds of meeting the ASES PASS cutoff (all *P* > .13).

Examining predictors of VAS outcomes at follow-up, we found that longer follow-up time was associated with lower (better) VAS scores (R^2^ = 2.4%; *P* = .01). Similarly, we found that longer follow-up time was also associated with higher odds of meeting the VAS PASS cutoff (OR, 1.6; *P* = .02). Specifically, for every 1-year increase in follow-up time, there was approximately 1.6 times the odds of meeting the VAS PASS cutoff. Sex, tear size, age at surgery, age at follow-up, and number of medial row anchors were not significantly associated with either VAS score nor the odds of meeting the VAS PASS cutoff (all *P* > .24). Table III shows a summary of factors significantly associated with higher/better ASES and VAS scores in those with all-suture anchors.

**Table III:** Summary of clinical factors significantly associated with higher/better outcomes.

Outcome	Medial row anchor group	
	All-suture anchors (n = 280)	Solid anchors (n = 72)
ASES score	Male sex Increased follow-up time	Smaller tear size (small vs. large tear)
ASES PASS	Male sex Increased follow-up time	Smaller tear size (small vs. large tear)
VAS score	Increased follow-up time	Smaller tear size (small vs. large tear)
VAS PASS	Increased follow-up time	Increased follow-up time

*ASES*, American Shoulder and Elbow Surgeons; *VAS*, visual analog scale; *PASS*, Patient Acceptable Symptomatic State.

### Predictors of outcomes: solid anchor group (n = 72)

Examining predictors of ASES outcomes at follow-up in the solid anchor group only, we found that large tears (in comparison to small tears) were associated with lower (worse) ASES scores (R2 [model] = 14.6%; *P* < .01). We also found that massive tears (in comparison to small tears) demonstrated a statistical trend toward association with lower (worse) ASES scores, but this did not reach statistical significance (R2 [model] = 14.6%; *P* = .07). Similarly, we found that large tears (in comparison to small tears) were associated with lower odds of meeting the ASES PASS cutoff (OR 0.11; *P* = .02). Specifically, having a large tear size vs. a small tear size associated with approximately 89% decreased odds of meeting the ASES PASS cutoff. ASES scores and the proportions of those meeting the ASES PASS cutoff at follow-up categorized by tear size are shown in Supplementary Appendix S2 (Supplemental Data). Interestingly, in the solid anchor group, patients with massive tears (n = 8) had better ASES scores, PASS cutoff percentage, and VAS scores than the group with large tears (n = 11) Sex, follow-up time, age at surgery, age at follow-up, and the number of medial anchors used were not significantly associated with ASES score or with the odds of meeting the ASES PASS cutoff at follow-up (all *P* > .11).

Examining predictors of VAS outcomes at follow-up, we found that we found that large tears (in comparison to small tears) were associated with higher (worse) VAS scores (R2 [model] = 13.0%; *P* < .01). We also found that massive tears (in comparison to small tears) demonstrated a statistical trend toward association with higher (worse) VAS scores, but this did not reach statistical significance (R2 [model] = 13.0%; *P* = .08). VAS scores at follow-up categorized by tear size are shown in Supplementary Appendix S2 (Supplemental Data). In addition, we found that longer follow-up time was associated with higher odds of meeting the VAS PASS cutoff (OR 2.4, *P* = .04). Specifically, for every 1-year increase in follow-up time, there was approximately 2.4 times the odds of meeting the VAS PASS cutoff. Table III shows a summary of factors significantly associated with improved ASES and VAS scores in those with solid anchors.

## Discussion

This study aimed to examine clinical factors associated with outcomes following arthroscopic double-row RCR performed with either solid or all-suture medial row anchors. There were no significant differences in function, as demonstrated by ASES score and the proportions of patients who met the ASES PASS cutoffs, or postoperative pain, as measured by the VAS and the proportions of patients meeting VAS PASS cutoffs, between suture anchor groups, demonstrating that all-suture anchors seem to perform equivalently to traditional solid anchors. Furthermore, more than three-quarters of patients achieved acceptable shoulder function (as measured by the ASES and VAS PASS cutoffs), regardless of anchor type utilized.

In patients who underwent RCR with all-suture anchors, longer follow-up time was found to be associated with higher (better) ASES scores, lower (better) VAS scores, and a higher likelihood of meeting both the ASES and VAS PASS cutoffs. In addition, male sex was associated with higher ASES score and a higher likelihood of meeting the ASES PASS cutoff. In patients who underwent RCR with solid anchors, smaller tear size was associated with higher ASES and VAS scores, as well as a higher likelihood of meeting the ASES PASS cutoff. In addition, longer follow-up time was associated with a higher likelihood of meeting the VAS PASS cutoff. Prior meta-analyses have shown that predictors of RCR outcomes are currently unclear or inconsistent at best.^37^ Our study suggests that predictors of clinical outcomes may be specific to anchor selection, emphasizing the importance of anchor selection as a potential physician-chosen factor in facilitating optimal longitudinal outcomes following RCR.

Longer follow-up time was shown to be associated with higher functional scores and lower pain in the all-suture anchor group in the present work. In a similar study evaluating longitudinal outcomes of arthroscopic double-row transosseous-equivalent RCR, Rakowski et al^13^ reported an average ASES score of 93.1 at an average of 11.5 years postoperatively but noted a statistically significant decline in scores as follow-up time increased. Their findings are similar to the average ASES score of 88.8 within our entire cohort. Nicholson et al^29^ also evaluated longitudinal outcomes of arthroscopic RCR and found that ASES scores were highest at two years postoperatively, with a modest decrease in scores at postoperative years 5 and 15. That study observed no relationship between VAS score and duration of follow-up time after 1 year postoperatively.^29^ In contrast to the findings in these prior studies, we observed that longer follow-up time was associated with higher ASES scores. However, the range of follow-up in our study was only between 2 and approximately 4.5 years. Taken together, these findings appear to suggest that functional and pain-related improvements after arthroscopic RCR stabilize after the initial postoperative period (approximately 1-3 years), and, in some cases, may begin to worsen in the long-term following RCR.

In our study, we found male sex to be associated with both higher ASES scores at follow-up as well as higher odds of meeting the ASES PASS cutoff. Male sex has consistently been reported as a consistent predictor of better postoperative outcomes following RCR, regardless of anchors, techniques, or follow-up time.^1^^,^^19^^,^^21^^,^^26^^,^^29^ In fact, female sex is consistently described as a predictor of poorer clinical outcomes throughout the literature across a variety of orthopedic procedures.^18^^,^^27^^,^^40^ The mechanisms driving differential outcomes between sexes is not currently clear, and further work is clearly needed to better understand the pathophysiology that predisposes women to poorer orthopedic outcomes, including poorer RCR outcomes, and to develop new clinical strategies to mitigate this risk.

Notably, in both the all-suture and solid anchor groups, our study found that age at the time of surgery was not associated with functional or pain-related outcomes (ASES or VAS), in contrast with findings described in similar studies. Multiple studies, including meta-analyses, have reported that increased age is a risk factor for failure of RCR healing, retear, and poorer postoperative outcomes.^7^^,^^29^^,^^32^^,^^37^ Specifically, in a study evaluating outcomes of all-suture anchor medial row RCR, older age was found to be associated with worsening VAS score; however, this study's follow-up time was only an average of 1-year postoperatively.^23^ Interestingly, Malavolta et al^21^ evaluated outcomes at 2 years postoperatively and found that older age was associated with improved outcomes. In addition, another meta-analysis found that older age compromised cuff integrity but had no effect on function-related outcomes.^31^ Oh et al previously reported that the integrity of the RCR, evaluated via computed tomographic arthrography, was not directly related to functional outcomes, including ASES and VAS scores. These findings support our observation that age was not associated with functional or pain-related outcomes following RCR in either anchor group.^30^

In the solid anchor group, large tear size, as compared to small tear size, was shown to be predictive of poorer outcomes. Similar findings have been reported previously, with larger tear size shown to be associated with higher risk of retear and poorer functional outcomes.^7^^,^^16^ Notably, the all-suture anchor demonstrated similar outcomes regardless of tear size, and tear size had no significant predictive association with outcomes. Memon et al^23^ evaluated short-term outcomes following RCR with a medial row all-suture anchor technique and similarly found that tear size was not associated with outcomes. These results may suggest that all-suture anchors and use of the medial row technique may offer equivalent fixation and functional outcomes across all rotator cuff tear sizes. Thus, all-suture anchors may help facilitate more comparable outcomes and prognosis across all RCR presentations, particularly in challenging clinical scenarios for the use of traditional anchors.

## Limitations

Our study has several important limitations. First, this study lacks radiographic follow-up data. Radiographic studies are not indicated in standard postoperative clinical care; however, without these data, it is not possible to make statements regarding the impact of anchor selection on longitudinal tissue integrity and joint health. Furthermore, our study did not collect data regarding medical comorbidities, such as diabetes, tobacco use, or connective tissue disorders, which may have important effects on RCR outcomes.^21^^,^^26^^,^^31^^,^^33^ However, the presence of comorbid conditions was not included in our exclusion criteria, so these conditions are likely present in our cohort in the same prevalence as they exist in the patient population, increasing generalizability of our findings. In addition, whereas all surgeons with patients in this study were fellowship-trained and used the same transosseous-equivalent (suture bridge) technique, not all surgeons switched to the use of medial row all-suture anchors at the same time, and all continued to occasionally use medial row solid anchors based on the individual patient's clinical scenario. Lastly, we did not collect data on concomitant distal clavicle excision or biceps tenodesis, and thus we were unable to evaluate for differences between anchor groups nor their effect on outcomes.

## Conclusion

This study demonstrates that all-suture anchor medial row RCRs perform at least as well as solid anchor medial row repairs with respect to mid-term patient-reported outcomes. Interestingly, despite similarity in outcomes between arthroscopic RCR performed with solid anchors or all-suture medial row anchors, predictors of longitudinal clinical outcomes did differ between anchor types. When all-suture anchors were used, male sex and longer follow-up time were associated with better postoperative outcomes. However, when solid anchors were used, smaller tear size was shown to be associated with better outcomes.

## Disclaimers:

Funding: This study was funded in part by support from Arthrex (Grant US-00697). The funding agency had no role in the collection, analysis, nor interpretation of the data presented herein. In addition, the funding agency had no involvement in the writing of the manuscript, nor the decision to submit the manuscript for publication.

Conflicts of interest: Marcus A. Rothermich, MD has received education payments from Arthrex, Smith & Nephew, and Zimmer Biomet and nonconsulting fees from Arthrex. Michael K. Ryan, MD has received grant support from Arthrex; education payments from Arthrex, DJO, Fones Marketing Management, and Smith & Nephew; consulting fees and speaking fees from Arthrex and Zimmer Biomet; and hospitality payments from Linvatec and Prime Surgical. Benton A. Emblom, MD has received consulting fees, nonconsulting fees, and royalties from Arthrex. Jeffrey R. Dugas, MD has received consulting fees from Arthrex, Bioventus, DJO, Royal Biologics, and Smith & Nephew; nonconsulting fees from Arthrex; royalty from Arthrex and In2Bones. E. Lyle Cain, MD has received education payments from Prime Surgical and Zimmer Biomet; consulting fees from Arthrex, DJO, Smith & Nephew, and Zimmer Biomet; nonconsulting fees from Arthrex, Medical Device Business Services, and Smith & Nephew; royalties from Arthrex; and hospitality payments from Encore Medical. The other authors, their immediate families, and any research foundation with which they are affiliated have not received any financial payments or other benefits from any commercial entity related to the subject of this article.
