# Supplementary material for: Predictors of outcomes following double-row rotator cuff repair: an assessment of all-suture or solid medial row anchor utilization at a single high-volume institution
Source: JSES Rev Rep Tech. 2025 Dec 11;6(2):100639. doi: 10.1016/j.xrrt.2025.100639 (PMC12887385; doi:10.1016/j.xrrt.2025.100639)
Supplement: Appendix 1 [file mmc1.docx]

**Appendix 1: Supplemental Data Showing Outcomes by Males and Females within the All-Suture Anchor Group (n=280)**

|  | **Males (n=172)** | **Females (n=108)** |
| --- | --- | --- |
| **ASES Score at Follow-Up** | 90.5 ± 15.1 | 86.2 ± 18.7 |
| **Proportion Meeting ASES PASS Cutoff at Follow-Up** | 84% | 75% |

ASES - American Shoulder and Elbow Surgeons Standardized Shoulder Assessment; PASS - Patient Acceptable Symptomatic State
