# Supplementary material for: Predictors of outcomes following double-row rotator cuff repair: an assessment of all-suture or solid medial row anchor utilization at a single high-volume institution
Source: JSES Rev Rep Tech. 2025 Dec 11;6(2):100639. doi: 10.1016/j.xrrt.2025.100639 (PMC12887385; doi:10.1016/j.xrrt.2025.100639)
Supplement: Appendix 2 [file mmc2.docx]

**Appendix 2: Supplemental Data Showing Outcomes by Tear Size within the Solid Anchor Group (n=72)**

|  | **Small (n=35)** | **Medium (n=18)** | **Large (n=11)** | **Massive (n=8)** |
| --- | --- | --- | --- | --- |
| **ASES Score at Follow-Up** | 94.7 ± 8.6 | 91.4 ± 14.6 | 76.4 ± 27.1 | 82.7 ± 27.7 |
| **Proportion Meeting ASES PASS Cutoff at Follow-Up** | 86% | 83% | 64% | 75% |
| **VAS Score at Follow-Up** | 0.6 ± 1.0 | 0.9 ± 1.5 | 2.6 ± 3.3 | 2.0 ± 3.7 |

ASES - American Shoulder and Elbow Surgeons Standardized Shoulder Assessment; PASS - Patient Acceptable Symptomatic State
